# 1,2-Diiodo-4,5-dimethyl­benzene

**DOI:** 10.1107/S1600536809016018

**Published:** 2009-05-07

**Authors:** Bruce A. Hathaway, Uriah J. Kilgore, Marcus R. Bond

**Affiliations:** a Department of Chemistry, Southeast Missouri State University, Cape Girardeau, Missouri 63701, USA

## Abstract

The structure of the title compound, C_8_H_8_I_2_, conforms closely to the *mm*2 symmetry expected for the free mol­ecule and is the first reported structure of a diiodo­dimethyl­benzene. Repulsion by neighboring I atoms and the neighboring methyl groups opposite to them results in a slight elongation of the mol­ecule along the approximate twofold rotation axis that bis­ects the ring between the two I atoms. In the extended structure, the mol­ecules form inversion-related pairs which are organized in approximately hexa­gonal close-packed layers and the layers then stacked so that mol­ecules in neighboring layers abut head-to-tail in a manner that optimizes dipole–dipole inter­actions.

## Related literature

For the synthesis see: Suzuki (1988 ▶). For the structure of 1,2-diiodo-4,5-dimethoxy­benzene, see: Cukiernik *et al.* (2008 ▶). For methods of iodinating substituted benzenes, see: Hathaway *et al.* (2007 ▶). For related work on diacetyl­enes, see: Hathaway (1988 ▶); Hathaway & Scates (1997 ▶).
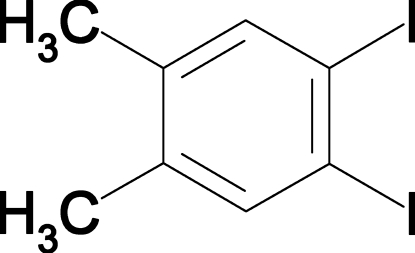


## Experimental

### 

#### Crystal data

C_8_H_8_I_2_
*M* *_r_* = 357.94
Monoclinic, 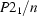

*a* = 9.4458 (1) Å
*b* = 8.1334 (1) Å
*c* = 13.4562 (2) Å
β = 110.109 (1)°
*V* = 970.77 (2) Å^3^
*Z* = 4
Mo *K*α radiation
μ = 6.41 mm^−1^
*T* = 298 K
0.3 × 0.2 × 0.18 mm

#### Data collection

Nonius KappaCCD diffractometer
Absorption correction: multi-scan (*SORTAV*; Blessing, 1995 ▶) *T*_min_ = 0.184, *T*_max_ = 0.316
32763 measured reflections
4243 independent reflections
2520 reflections with *I* > 2σ(*I*)
*R*_int_ = 0.057

#### Refinement

*R*[*F* ^2^ > 2σ(*F* ^2^)] = 0.035
*wR*(*F* ^2^) = 0.090
*S* = 1.10
4243 reflections
94 parameters
H-atom parameters constrained
Δρ_max_ = 0.90 e Å^−3^
Δρ_min_ = −0.75 e Å^−3^

### 

Data collection: *COLLECT* (Hooft, 1998 ▶); cell refinement: *SCALEPACK* (Otwinowski & Minor, 1997 ▶); data reduction: *SCALEPACK* and *DENZO* (Otwinowski & Minor, 1997 ▶); program(s) used to solve structure: *SHELXS97* (Sheldrick, 2008 ▶); program(s) used to refine structure: *SHELXL97* (Sheldrick, 2008 ▶); molecular graphics: *ORTEP-3* (Farrugia, 1997 ▶); software used to prepare material for publication: *WinGX* (Farrugia, 1999 ▶).

## Supplementary Material

Crystal structure: contains datablocks global, I. DOI: 10.1107/S1600536809016018/is2407sup1.cif

Structure factors: contains datablocks I. DOI: 10.1107/S1600536809016018/is2407Isup2.hkl

Additional supplementary materials:  crystallographic information; 3D view; checkCIF report

## Figures and Tables

**Fig. 1.**
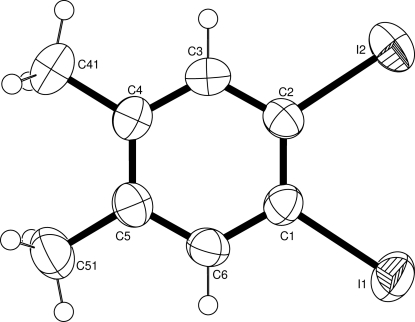
View of (I) (50% probability displacement ellipsoids).

**Fig. 2.**
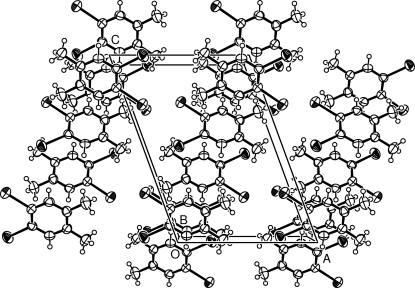
Packing diagram of the structure, viewed down the b axis.

## supplementary crystallographic information

### Comment

1,2-Diiodo-4,5-dimethylbenzene, (I), was prepared as part of an investigation of methods of iodination of organic compounds. In previous work we prepared diacetylenes as potential non-linear optical materials (Hathaway, 1988; Hathaway & Scates, 1997). Since iodobenzenes are common starting materials for preparations of phenyl alkynes, we investigated several methods of iodinating substituted benzenes (Hathaway *et al.*, 2007). The title compound was prepared as an extension of this investigation. Ortho-xylene was iodinated using iodine and periodic acid (Suzuki, 1988), to learn if two iodine atoms could be substituted onto the benzene ring in the positions opposite the methyl groups. Since no structures of diiododimethylbenzenes have yet been reported, we undertook the crystal structure determination of (I). The structure of the similar compound 1,2-diiodo-4,5-dimethoxybenzene has recently been reported. (Cukiernik *et al.*, 2008). The structure of (I) offers a distinct contrast due to the lack of key intermolecular interactions.

Although the title molecule does not possess any crystallographic site symmetry, it closely approximates *mm*2 point symmetry– even by the methyl hydrogen atoms (which were clearly identified on an electron density difference map). The two C—I bonds have lengths [2.097 (3) Å] that are identical to each other and also agree with the sum of the covalent radii within 1 su. Carbon-carbon bond lengths and angles within the aromatic ring and to the methyl groups conform closely to expected values. The average C—C bond length within the ring is 1.387 Å with deviations from the average within 1 su. Steric repulsion between the iodine atoms *ortho* to one another on the ring increases the facing C—C—I angles by over 3° from the ideal value. A similar repulsive distortion between the methyl groups *ortho* to one another is also found to give facing C—C—C angles between 121 and 122°. Internal C—C—C angles for the aromatic ring are greater than 120° for the two ring atoms without substituents (C3 and C6) and are less than 120° for the substituted ring atoms. This arises from a slight elongation of the ring along the approximate twofold rotation axis that bisects ring the between like substituent groups. The elongation produces contact distances to the opposite atom in the ring that are longer [2.791 (6) Å] for subsituted ring atoms than for the two unsubstituted ring atoms [2.738 (5) Å]. The I1—C1—C2—I2 and C41—C4—C5—C51 torsion angles are similar in value [2.5 (4) and 2.6 (6) ° respectively] and arise from just a small twist of the molecular plane about its long axis. A thermal ellipsoid plot of the molecule is presented in Figure 1.

The molecules pack in layers coinciding with the (101) family of planes. The layers are constructed from pairs of inversion related molecules which have their iodine atoms pointing in opposite directions outward from the the layer plane. The perpendicular distance between the molecular planes of the inversion related molecules is 3.752 (12) Å, although the molecules are displaced so that only the C2 and C3 atoms of each ring overlap the other ring completely. The long axis of the molecule is almost perpendicular to the *b* axis (within 3°) so, since all molecules in the crystal can be related to one another by inversion, *n*-glide, or lattice translation operations, molecular axes of all molecules are almost parallel (if not exactly parallel) to one another and tilted at an angle of 16.9 (3)° with respect to the normal to the layer plane. The molecular plane does form an angle of 30.87 (7)° with respect to the *b* axis, so molecules related by an *n*-glide operation take alternating orientations with respect to the *b* axis. This canting of the molecular planes leads to the shortest I···I contact distance within the layer, 4.1737 (3) [I1···I2^i^; symmetry code: (i) *x* - 1/2,3/2 - *y*,*z* - 1/2]. Inversion related pairs are hexagonal close packed to form the layer. Nearest neighbor pairs are related by a *b* axis translation or an *n*-glide operation yielding slightly different distances [8.1334 (1) Å and 7.8906 (1) Å, respectively] between neighboring inversion centers and a small distortion from an ideal hcp arrangement.

Neighboring layers can be related to one another by an *a* axis translation. The *a* axis translation does establish a head-to-tail line of molecules in which the diiodo end of a molecule in one layer abuts the dimethyl end of a molecule in a neighboring layer and *vice versa*. The shortest I···H contact distances both within and between layers range from 3.32–3.35 Å and are close to the sum of the van der Waals radii. So it is unlikely that significant hydrogen-bonding exists in the stucture. A short I···I interaction, 4.2126 (4) Å [I1···I2^ii^; symmetry code: (ii) -*x* + 3/2,*y* - 1/2,-*z* + 1/2], is found between molecules in different layers and related to one another by a 2_1_ rotation. These short intra- and inter-layer I···I contact distances are comparable in length to those found in the 1,2-diiodo-4,5-dimethoxy structure and in both structures do play a role in the packing arrangement. However the inversion-pairing within layers and head-to-tail arrangement between layers of molecules in (I) is more simply explained on the basis of optimizing dipole-dipole interactions. In contrast, the presence of I···O contacts in the dimethoxy analogue dictate a more complicated packing arrangement based on linear chains of molecules. The simpler structure of (I) in the absence of any I···O contacts further supports the earlier conclusion that these I···O contacts prevail in establishing the molecular packing in the dimethoxy analogue. A unit cell packing diagram of the structure is shown in Figure 2.

### Experimental

Iodine (45.72 g, 0.18 mol), periodic acid dihydrate (13.7 g, 0.060 mol) and *ortho*-xylene (22.3 g, 0.21 mol) were combined in a round-bottomed flask. To this mixture, a solution of 6 ml of concentrated sulfuric acid, 40 ml of water and 200 ml of glacial acetic acid was added. The resulting purple solution was refluxed overnight. The reaction mixture was cooled to room temperature, and water was added to precipitate a purple solid. The solid was collected, washed with water, and recrystallized from acetone to yield 40.5 g (63.2%) of the title compound, melting point 90–92 °C.

^1^H-NMR (CDCl_3_, 300 MHz): δ 7.62 (s, 2H, aromatic H's), 2.16 (s, 6H, CH_3_'s).

### Refinement

All hydrogen atoms were clearly visible on a difference Fourier map but, because of the heavy atoms present, their positions were calculated to give an idealized geometry, with C—H bond distances of 0.96 Å for methyl hydrogen atoms and 0.93 Å for aromatic hydrogen atoms. They were constrained to ride on their parent carbon atoms during refinement with the torsion angle for the methyl hydrogen atoms refined to best match the observed electron density.

### Crystal data

**Table:**

C_8_H_8_I_2_	*F*(000) = 648
*M**_r_* = 357.94	*D*_x_ = 2.449 Mg m^−^^3^
Monoclinic, *P*2_1_/*n*	Melting point = 365–363 K
Hall symbol: -P 2yn	Mo *K*α radiation, λ = 0.71073 Å
*a* = 9.4458 (1) Å	Cell parameters from 4493 reflections
*b* = 8.1334 (1) Å	θ = 2.9–35.0°
*c* = 13.4562 (2) Å	µ = 6.41 mm^−^^1^
β = 110.109 (1)°	*T* = 298 K
*V* = 970.77 (2) Å^3^	Prism, colorless
*Z* = 4	0.3 × 0.2 × 0.18 mm

### Data collection

**Table:**

Nonius KappaCCD diffractometer	2520 reflections with *I* > 2σ(*I*)
φ and ω scans	*R*_int_ = 0.057
Absorption correction: multi-scan (*SORTAV*; Blessing, 1995)	θ_max_ = 35.1°, θ_min_ = 3.0°
*T*_min_ = 0.184, *T*_max_ = 0.316	*h* = −15→15
32763 measured reflections	*k* = −13→13
4243 independent reflections	*l* = −21→21

### Refinement

**Table:**

Refinement on *F*^2^	H-atom parameters constrained
Least-squares matrix: full	*w* = 1/[σ^2^(*F*_o_^2^) + (0.0253*P*)^2^ + 1.2156*P*] where *P* = (*F*_o_^2^ + 2*F*_c_^2^)/3
*R*[*F*^2^ > 2σ(*F*^2^)] = 0.035	(Δ/σ)_max_ = 0.002
*wR*(*F*^2^) = 0.090	Δρ_max_ = 0.90 e Å^−^^3^
*S* = 1.10	Δρ_min_ = −0.75 e Å^−^^3^
4243 reflections	Extinction correction: *SHELXL97* (Sheldrick, 2008), Fc^*^=kFc[1+0.001xFc^2^λ^3^/sin(2θ)]^-1/4^
94 parameters	Extinction coefficient: 0.0069 (6)
0 restraints	

### Special details

**Table:**

Experimental. 13 C-NMR (CDCl3, 75.5 MHz): δ 140.0, 138.6, 104.0, 19.1. IR (thin film on a KBr disk) cm-1: 2976, 2938, 1720, 1438, 1370, 1329, 1264, 1153, 1018, 864.
Geometry. All e.s.d.'s (except the e.s.d. in the dihedral angle between two l.s. planes) are estimated using the full covariance matrix. The cell e.s.d.'s are taken into account individually in the estimation of e.s.d.'s in distances, angles and torsion angles; correlations between e.s.d.'s in cell parameters are only used when they are defined by crystal symmetry. An approximate (isotropic) treatment of cell e.s.d.'s is used for estimating e.s.d.'s involving l.s. planes.Mean plane calculation for molecule: m1 = -0.09103(0.00034) m2 = 0.84014(0.00006) m3 = -0.53468(0.00010) D = 1.69893(0.00184) Atom d s d/s (d/s)**2 I1 * -0.0006 0.0003 - 1.888 3.565 I2 * 0.0008 0.0003 2.741 7.511 C1 * -0.0032 0.0034 - 0.919 0.844 C2 * -0.0310 0.0033 - 9.414 88.619 C3 * -0.0569 0.0035 - 16.075 258.411 C4 * -0.0270 0.0036 - 7.562 57.183 C5 * 0.0431 0.0036 11.962 143.098 C6 * 0.0336 0.0037 9.187 84.400 C41 * -0.0843 0.0048 - 17.682 312.643 C51 * 0.1187 0.0049 24.074 579.539 ============ Sum((d/s)**2) for starred atoms 1535.813 Chi-squared at 95% for 7 degrees of freedom: 14.10 The group of atoms deviates significantly from planarity

### Fractional atomic coordinates and isotropic or equivalent isotropic displacement parameters (Å^2^)

**Table:**

	*x*	*y*	*z*	*U*_iso_*/*U*_eq_	
I1	0.60697 (3)	0.54314 (4)	0.23538 (2)	0.06585 (12)	
I2	0.74073 (3)	0.78802 (4)	0.48114 (2)	0.06329 (11)	
C1	0.4696 (4)	0.6067 (4)	0.3230 (3)	0.0443 (7)	
C2	0.5208 (4)	0.6956 (4)	0.4163 (3)	0.0432 (7)	
C3	0.4222 (4)	0.7288 (4)	0.4696 (3)	0.0472 (7)	
H3	0.4573	0.7864	0.5331	0.057*	
C4	0.2729 (4)	0.6790 (4)	0.4315 (3)	0.0465 (7)	
C5	0.2204 (4)	0.5941 (4)	0.3360 (3)	0.0478 (7)	
C6	0.3202 (4)	0.5567 (5)	0.2835 (3)	0.0484 (8)	
H6	0.2861	0.497	0.2208	0.058*	
C41	0.1722 (5)	0.7162 (6)	0.4942 (4)	0.0655 (11)	
H41A	0.0932	0.7893	0.4546	0.098*	
H41B	0.2302	0.7671	0.56	0.098*	
H41C	0.1289	0.6158	0.5082	0.098*	
C51	0.0575 (5)	0.5410 (6)	0.2889 (4)	0.0686 (11)	
H51A	0.0313	0.476	0.3395	0.103*	
H51B	0.0438	0.477	0.2263	0.103*	
H51C	−0.0061	0.6364	0.2708	0.103*	

### Atomic displacement parameters (Å^2^)

**Table:**

	*U*^11^	*U*^22^	*U*^33^	*U*^12^	*U*^13^	*U*^23^
I1	0.06034 (17)	0.0866 (2)	0.05987 (17)	0.00565 (14)	0.03258 (13)	−0.00308 (14)
I2	0.04658 (14)	0.06653 (19)	0.06980 (19)	−0.01353 (11)	0.01107 (11)	−0.00086 (13)
C1	0.0471 (16)	0.0459 (17)	0.0444 (16)	−0.0011 (14)	0.0215 (13)	0.0011 (13)
C2	0.0424 (15)	0.0438 (17)	0.0406 (15)	−0.0035 (13)	0.0106 (12)	0.0038 (13)
C3	0.0541 (18)	0.0462 (18)	0.0394 (15)	−0.0009 (15)	0.0137 (13)	−0.0036 (14)
C4	0.0478 (17)	0.0467 (18)	0.0489 (17)	0.0030 (14)	0.0217 (14)	0.0042 (14)
C5	0.0437 (16)	0.0456 (18)	0.0529 (18)	−0.0012 (14)	0.0152 (14)	0.0045 (14)
C6	0.0485 (18)	0.0529 (19)	0.0411 (16)	−0.0062 (15)	0.0119 (13)	−0.0046 (14)
C41	0.068 (3)	0.075 (3)	0.067 (2)	0.005 (2)	0.041 (2)	0.000 (2)
C51	0.046 (2)	0.073 (3)	0.083 (3)	−0.007 (2)	0.0168 (19)	−0.006 (2)

### Geometric parameters (Å, °)

**Table:**

I1—C1	2.097 (3)	C41—H41B	0.96
I2—C2	2.097 (3)	C41—H41C	0.96
C1—C2	1.384 (5)	C4—C5	1.391 (5)
C1—C6	1.386 (5)	C5—C51	1.512 (5)
C2—C3	1.384 (5)	C51—H51A	0.96
C3—C4	1.385 (5)	C51—H51B	0.96
C3—H3	0.93	C51—H51C	0.96
C4—C41	1.504 (5)	C5—C6	1.392 (5)
C41—H41A	0.96	C6—H6	0.93
			
I1···I2^i^	4.1737 (3)	I1···I2^ii^	4.2126 (4)
			
C2—C1—C6	119.6 (3)	H41A—C41—H41C	109.5
C2—C1—I1	123.2 (2)	H41B—C41—H41C	109.5
C6—C1—I1	117.2 (2)	C5—C4—C41	121.7 (4)
C1—C2—C3	119.0 (3)	C4—C5—C6	119.2 (3)
C1—C2—I2	123.5 (2)	C4—C5—C51	121.2 (4)
C3—C2—I2	117.5 (2)	C5—C51—H51A	109.5
C2—C3—C4	122.1 (3)	C5—C51—H51B	109.5
C2—C3—H3	118.9	H51A—C51—H51B	109.5
C4—C3—H3	118.9	C5—C51—H51C	109.5
C3—C4—C5	118.8 (3)	H51A—C51—H51C	109.5
C3—C4—C41	119.5 (3)	H51B—C51—H51C	109.5
C4—C41—H41A	109.5	C6—C5—C51	119.6 (4)
C4—C41—H41B	109.5	C1—C6—C5	121.3 (3)
H41A—C41—H41B	109.5	C1—C6—H6	119.4
C4—C41—H41C	109.5	C5—C6—H6	119.4

Symmetry codes:  (i) *x*−1/2, −*y*+3/2, *z*−1/2; (ii) −*x*+3/2, *y*−1/2, −*z*+1/2.
